# HCV transmission in high-risk communities in Bulgaria

**DOI:** 10.1371/journal.pone.0212350

**Published:** 2019-03-05

**Authors:** Lilia Ganova-Raeva, Zoya Dimitrova, Ivailo Alexiev, Lili Punkova, Amanda Sue, Guo-liang Xia, Anna Gancheva, Reneta Dimitrova, Asya Kostadinova, Elitsa Golkocheva-Markova, Yury Khudyakov

**Affiliations:** 1 Centers for Disease Control and Prevention, Division of Viral Hepatitis, Molecular Epidemiology and Bioinformatics, Atlanta, GA, United States of Ameirca; 2 National Reference Confirmatory Laboratory for HIV, National Center for Infectious and Parasitic Diseases, Sofia, Bulgaria; 3 National Reference Laboratory of Hepatitis, National Center for Infectious and Parasitic Diseases, Sofia, Bulgaria; University of Cincinnati College of Medicine, UNITED STATES

## Abstract

**Background:**

The rate of HIV infection in Bulgaria is low. However, the rate of HCV-HIV-coinfection and HCV infection is high, especially among high-risk communities. The molecular epidemiology of those infections has not been studied before.

**Methods:**

Consensus Sanger sequences of HVR1 and NS5B from 125 cases of HIV/HCV coinfections, collected during 2010–2014 in 15 different Bulgarian cities, were used for preliminary phylogenetic evaluation. Next-generation sequencing (NGS) data of the hypervariable region 1 (HVR1) analyzed via the Global Hepatitis Outbreak and Surveillance Technology (GHOST) were used to evaluate genetic heterogeneity and possible transmission linkages. Links between pairs that were below and above the established genetic distance threshold, indicative of transmission, were further examined by generating k-step networks.

**Results:**

Preliminary genetic analyses showed predominance of HCV genotype 1a (54%), followed by 1b (20.8%), 2a (1.4%), 3a (22.3%) and 4a (1.4%), indicating ongoing transmission of many HCV strains of different genotypes. NGS of HVR1 from 72 cases showed significant genetic heterogeneity of intra-host HCV populations, with 5 cases being infected with 2 different genotypes or subtypes and 6 cases being infected with 2 strains of same subtype. GHOST revealed 8 transmission clusters involving 30 cases (41.7%), indicating a high rate of transmission.

Four transmission clusters were found in Sofia, three in Plovdiv, and one in Peshtera. The main risk factor for the clusters was injection drug use. Close genetic proximity among HCV strains from the 3 Sofia clusters, and between HCV strains from Peshtera and one of the two Plovdiv clusters confirms a long and extensive transmission history of these strains in Bulgaria.

**Conclusions:**

Identification of several HCV genotypes and many HCV strains suggests a frequent introduction of HCV to the studied high-risk communities. GHOST detected a broad transmission network, which sustains circulation of several HCV strains since their early introduction in the 3 cities. This is the first report on the molecular epidemiology of HIV/HCV coinfections in Bulgaria.

## Introduction

The rates of newly diagnosed infections with Human Immunodeficiency Virus (HIV) in Bulgaria are low; however, among individuals with HIV, the prevalence of coinfection with hepatitis B virus (HBV) and hepatitis C virus (HCV) and HIV coinfections fall within the upper range reported in Europe—10.4% and 25.6%, respectively [1, 2]. High rates of active hepatitis infections were confirmed by detection of HBV DNA in 51.1% and HCV RNA in 78.1% of the tested HIV-positive individuals [2]. Hepatitis virus coinfections with HIV affected mostly high-risk behavior groups, including people who inject drugs (PWID), men who have sex with men (MSM), people with history of incarceration, and Roma people [3].

HCV is genetically diverse and classified into 7 genotypes and 67 subtypes according to the National Center for Biotechnology information and peer reviewed sources [4]. Within each individual(intra-host), the infecting HCV strain forms a large population of closely related but distinct genetic variants, referred to as a quasispecies [5, 6], a feature that can be used to identify HCV strains [7]. In immune suppressed persons, such as those co-infected with HIV, intra-host diversity may decrease [8, 9]. However, intra-host HCV diversity is increased in persons with high-risk behaviors, in particular, unsafe injection drug use, owing to their frequent exposure to different HCV strains [10, 11]. Genetic analysis of HCV intra-host variants is important for the identification of HCV strains and transmission events [12]. The HCV hypervariable region 1 (HVR1) is frequently used for assessing HCV diversity [13, 14]. Measuring genetic distances among intra-host populations from infected persons accurately detects HCV transmissions [15].

Next-Generation Sequencing (NGS) allows for accurate characterization of intra-host HCV populations, detecting low frequency variants and rare mutations [16]. Application of amplicon-based NGS of intra-host HCV HVR1 variants [15] to molecular surveillance and outbreak investigation is daunting [17], owing to intra-host HCV genetic diversity and contribution of sequencing errors to the heterogeneity [18]. NGS generates large numbers of sequence reads from each patient, making visualization of phylogenetic analysis from many patients cumbersome and hard to interpret for the detection of transmissions in outbreak settings. Here, we take advantage of the Global Hepatitis Outbreak and Surveillance Technology (GHOST) to detect HCV transmission and visualize transmission networks in Bulgaria. GHOST uses unique haplotypes to measure genetic distance between populations of intra-host HCV HVR1 variants sequenced from each patient and generates a linkage network, where patients are linked if genetic distance between the HCV populations is below an empirically derived threshold [19, 20].

This study was aimed at using GHOST to conduct genetic surveillance of HCV infections among a population of HCV-HIV coinfected patients using serum specimens collected from HIV incidence cases identified during 2010–2014 in 15 cities in Bulgaria. As this is a cohort with high risk behavior the analyses were intended to gain insight into the variability of the HCV virus and any potential transmissions in circumstance when the disease incidence itself did not alert to an outbreak situation. This is the first report on the molecular epidemiology of HIV/HCV coinfections in the country.

## Materials and methods

### Specimens

**Fig 1:**
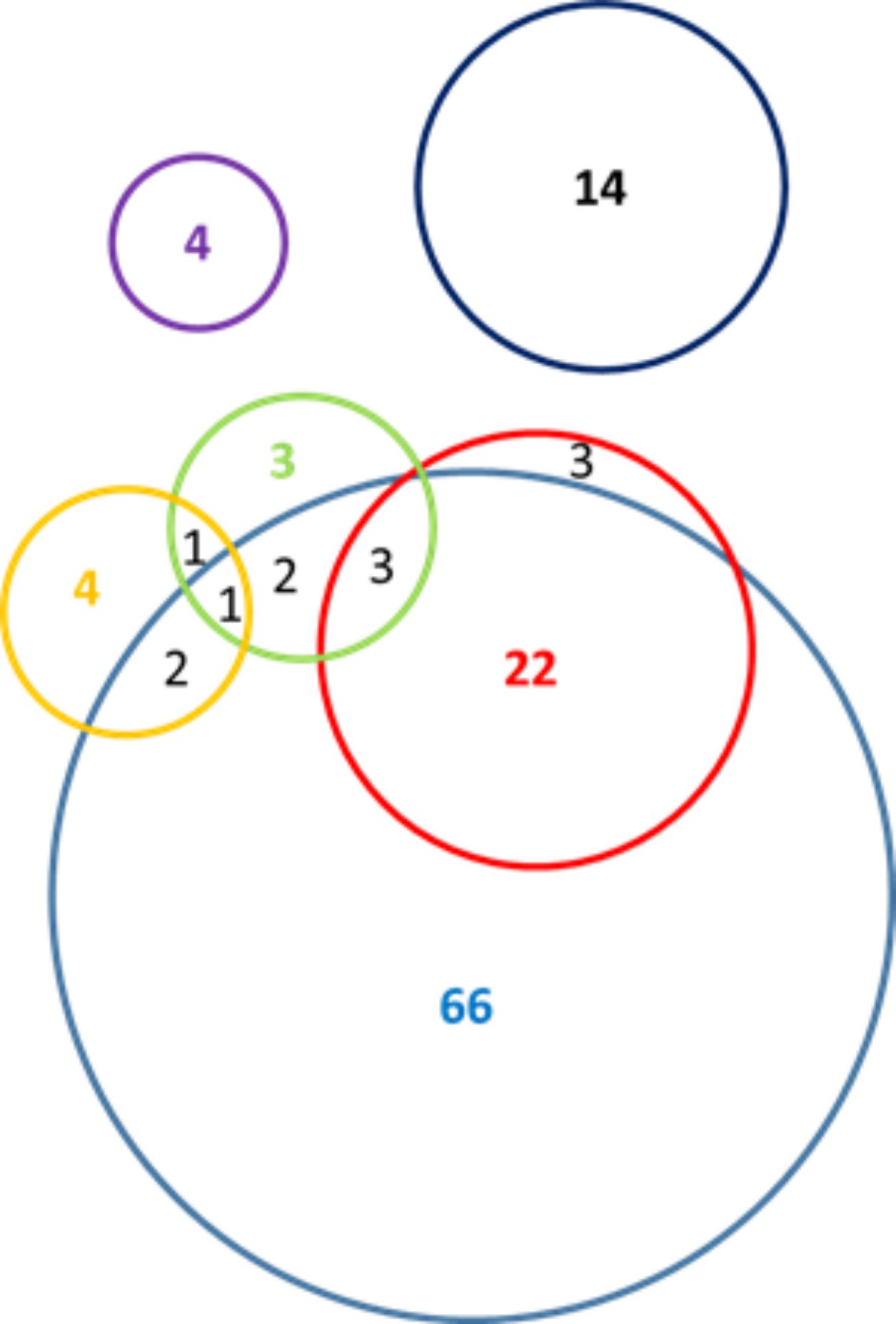
Venn diagram of risk factors among tested individuals (n = 125). PWID (n = 96)—blue, MSM (n = 10)—green, Incarceration (n = 28)—red, Blood transfusion (n = 4)—purple, Sex Workers (n = 8)—yellow, No reported risk (n = 14)—black.

Serum samples were available from 53.8% (n = 503) of all newly diagnosed HIV cases (n = 934) identified during 2010–2014 in Bulgaria [2]. All patients provided written informed consent to participate in this study and answered a questionnaire about sex, age, origin of birth, risk behaviors, partners (spouse or cohabitation), occupation, and if they have ever traveled to a foreign country, used injection drug, received blood transfusion or been imprisoned. The Ethics Committee at the National Center of Infectious and Parasitic Diseases, Sofia, Bulgaria (NCIPD IRB IORG00006384), approved the study. The specimens were tested for HCV antibody (anti-HCV) by commercial enzyme immunoassay kits (NanbaseC-96 3.0, General Biological Corporation, Hsin-Chu, Taiwan and Murex anti-HCV, version 4.0 Abbott/Murex, Murex Biotech South Africa Proprietary, Kyalami, Republic of South Africa). Anti-HCV positive samples (n = 203) were tested for HCV RNA using Amplicor Hepatitis C virus Test, version 2.0 (Roche Molecular Systems, Branchburg, New Jersey, USA) [2]. HCV RNA positive specimens (n = 171) were genotyped in this study (n = 125) and were used for further analysis. Fig 1 shows the distribution of risk factors among cases from 15 Bulgarian cities. Most individuals resided in Sophia (n = 59), Plovdiv (n = 41) and Peshtera (n = 6).

### Targeted amplification and library preparation

Total nucleic acid (TNA) was extracted from all specimens using 200 μl of serum on MagnaPureLC with TNA kit (Roche, Indianapolis, IN). The TNA was eluted in 50 μl and used for reverse transcription with random hexamers and SuperScript III enzyme, Vilo kit (Invitrogen, ThermoFisher, Waltham, MA). The cDNA was used to amplify fragments of NS5B (nt 8244–8645) and HVR1 (nt 1302–1610) as previously described [21]. In the case of HVR1, all specimens were labeled using unique 10-mer barcodes in both the reverse and forward primers. The amplicons were then indexed with 8-mer indexes and adapters (IDT) appropriate for Illumina sequencing, clustering and demultiplexing. All barcode and index sequences are listed in S1 Table. The products were purified using Ampure XP (Agilent) and quantified on Tape Station Instrument (Agilent, Santa Clara, CA) as directed by the manufacturers. The quantified amplicons were normalized and mixed to generate a 96 –multiplex library including 6 negative controls. The library was diluted to 10 pmol and sequenced using paired-end read protocol on Illumina MiSeq and v3 600 cycle chemistry. The library was automatically de-multiplexed on the MiSeq instrument.

### Sequence data

Purified NS5B and HVR1 amplicons were sequenced using BDv3.1 chemistry. The Sanger consensus contigs were assembled and analyzed with the DNASTAR Lasergene 14 package (SeqManPro, MegAlign, Madison, WI). Phylogenetic trees from the Sanger data were built using MEGA6 [22].

The paired demultiplexed Illumina reads were processed with GHOST [23]. The quality control module is a pipeline that includes multiple steps of data filtering, such as fragment length restrictions, majority barcode matching, matching of R1 and R2 overlapping sequence, uninterrupted ORF and quality threshold cut off [15, 23]. From the reads that passed all filters, 20,000 per specimen were randomly selected and the mismatches between R1 and R2 sequences were resolved. The unique haplotypes and their frequencies were determined and genotyped [23].

All haplotypes with a frequency of five or more were selected for further analysis of transmissions links, quasispecies network visualization and phylogenetic trees (GHOST, CLC Genomics workbench10.0.3, QIAGEN Aarhus A/S).

### Transmission links

Transmission links between cases were examined by the transmission detection module of GHOST. For each pair of cases, the populations were compared and the distance between them was defined as the Hamming distance between their closest haplotypes. If the distance was smaller than the empirically defined threshold of 0.037 [20, 23], these two cases were considered linked by transmission.

### K-step networks

To visualize the of inter-host HCV relatedness, k-step networks were created from the available NGS variants with a frequency of 5 or more [15]. The unique haplotypes were aligned and the Hamming distance between each haplotype pair was calculated. Then a one-step network was generated where each unique haplotype is represented by a node and two nodes were connected by an edge if the distance between them was 1. The network consisted of several connected components. To join the components together, k-step networks were constructed as follows: iteratively, for k = 2, 3, …, n, all pairs of haplotypes from different components with distance equal to k were found. They were linked by edges and the connected components were recalculated. If these steps were repeated until a single connected component was formed, the resulting network was equivalent to the union of all minimum spanning trees. The analysis and network visualization were performed using MATLAB R2017a (The Math Works, Inc.) and GEPHI. Links between pairs that were below and above the established genetic distance threshold of 0.037 [15], indicative of transmission, were further examined by generating and visualizing smaller k-step networks.

## Results

### Phylogenetic structure of the HCV population

Genotypes of the HCV strains (n = 125) were determined using phylogenetic analysis of the NS5b and HVR1 sequences obtained by Sanger sequencing. Genotype 1a was predominant (54%), followed by 1b (20.8%), 2a (1.4%), 3a (22.3%) and 4a (1.4%). For four cases, there was a discrepancy of the detected genotype between the two regions: cases 1513, 1216 and 1062 were genotyped as 1a or 1b, 1006 was genotyped as 1a or 4a using HVR1 and NS5B regions, respectively. For 1216, the availability of an additional specimen allowed for repeat testing to confirm the mixed infection finding (Table 1). Table 1 provides information about additional mixed infections detected by NGS of HVR1. The NS5B genotype was used to represent genotypic frequency in the population in cases where there was no HVR1 data available or there was a discrepancy between the calls.

**Table 1:** Characteristics of cases with mixed genotype infections as identified by NGS data. Case 1216 was confirmed 1a and 1b by Sanger sequencing.

	Gen (%)	Gen (%)	Risk factor	Gender	City
793	1b (71)	1a (29)	IDU	female	Plovdiv
833	1b (73.1)	1a (26.9)	Foreign travel	male	Sofia
918	1a (99.5)	3a (0.5)	IDU	female	Plovdiv
1216	1a (69.3)	1b (30.7)	None reported	male	Plovdiv
1438	4d (94.4)	3a (0.6)	IDU, Foreign travel	female	Blagoevgrad
704	3a (76.8)	3a (23.15)*	Foreign travel	female	Burgas
989	3a (91.2)	3a (9.8) *	None reported	male	Lovech
1207	1a (88.8)	1a (11.2)*	None reported	female	Plovdiv
1260	1a (56.4)	1a (43.6)*	MSM	male	Sofia
1356	3a (52.6)	3a (47.4)*	IDU	male	Burgas
1508	1a(69.3)	1a (30.7)*	Foreign travel	female	Plovdiv

*indicates superinfection with different strain from the same genotype.

**Fig 2:**
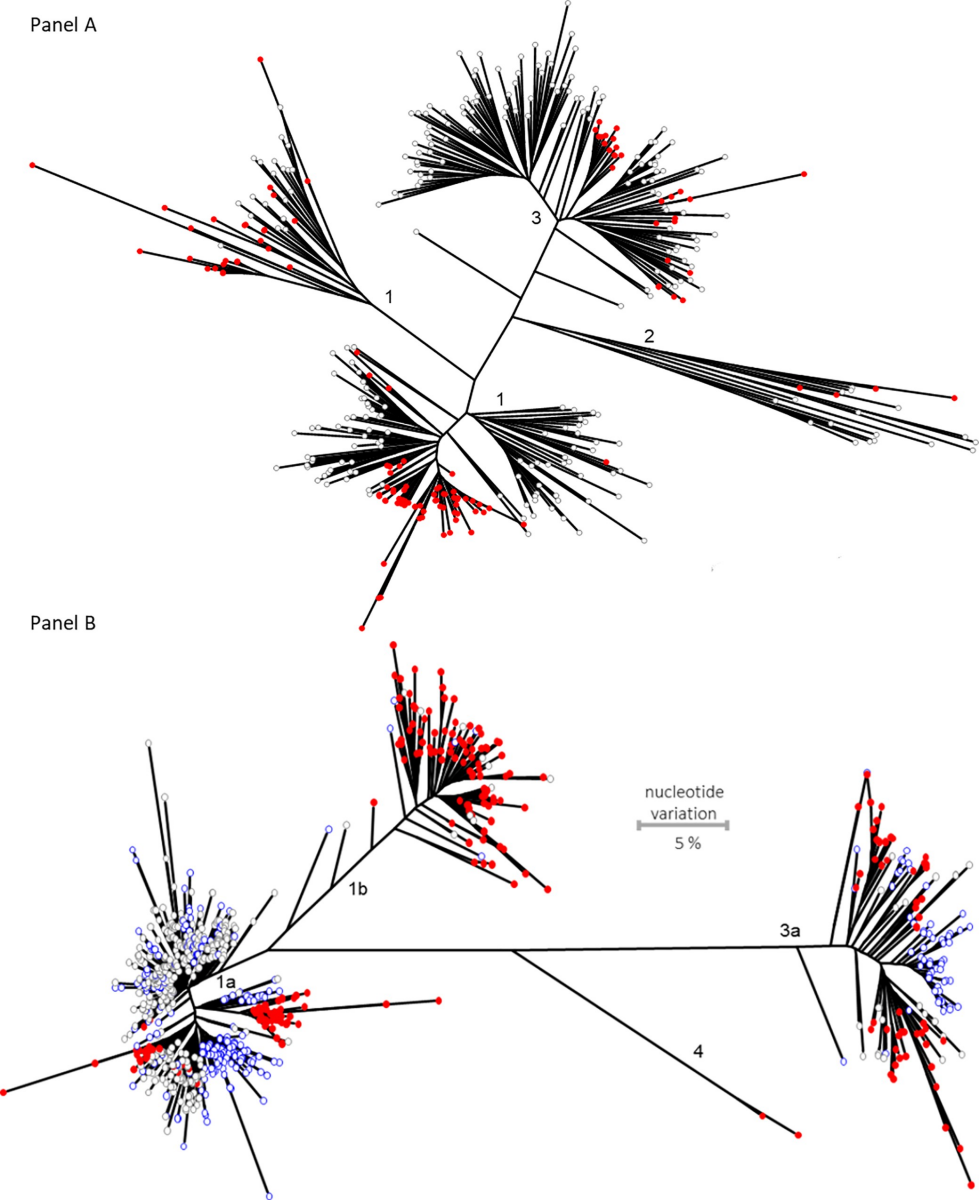
Phylogenetic analysis of NS5B (A) and HVR1 (B) Sanger sequences. Sequences obtained in this study (red) are shown in the background of sequences from North America (gray and blue). Bootstrap values for the major clusters were >80. Scale is the same for both panels. (A). NS5B phylogeny. (B). HVR1 Phylogeny.

The Bulgarian HVR1 and NS5B sequences formed 2–3 discernable monophyletic clusters in each of the major HCV subtypes, 1a, 1b and 3a (Fig 2). The largest HVR1 cluster involved >85% of all subtype 1a sequences generated here. These observations indicated that the studied high-risk population experienced several independent HCV introductions in the past. The ancestral strain for the largest cluster was most probably one of the first to be introduced in this population, suggesting that the HCV subtype 1a established itself earlier than any other HCV strain. Presence of long tips in the NS5B clusters of HCV (Fig 2) indicates a potentially long history of clusters in the population; while identification of many tightly related NS5B sequences in the clusters suggests a recent expansion of the HCV 1a strains.

### Intra-host genetic heterogeneity

The NGS yielded 10,083,029 reads, from 90 PCR positive cases with a sufficient testing volume, for an average of 112,034 reads per case (range 10,611–319,550). These data were processed by GHOST quality control pipeline with an output for 72 cases. After the random sampling of 20,000 reads for analysis, the average number of error-corrected reads per case was 16,342, ranging from 542–18,843. The average number of haplotypes was 7,362, ranging from 22–8,908.

### Mixed strain infections

The NGS data confirmed the genotypes obtained by Sanger sequencing. The 5 cases where we noted genotype discrepancies were resolved by NGS. For example, for case 793, Sanger sequencing of NS5b subtyped it as 1a and Sanger sequencing HVR1 revealed 1b. NGS data revealed the presence of both genotypes in this patient. The same was observed for the other cases shown in Table 1.

Another 6 cases, 704, 989, 1207, 1260, 1356, and 1508, were found to be infected with an additional strain of the same genotype, which contributed to increase in intra-host HCV genetic diversity, ranging from 0.05–0.089. Thus, the NGS data show that 15.3% of cases (n = 11) are infected with >1 HCV strain, indicating frequent co- or super-infections of members of the studied population with HCV strains.

### Transmission clusters

**Fig 3:**
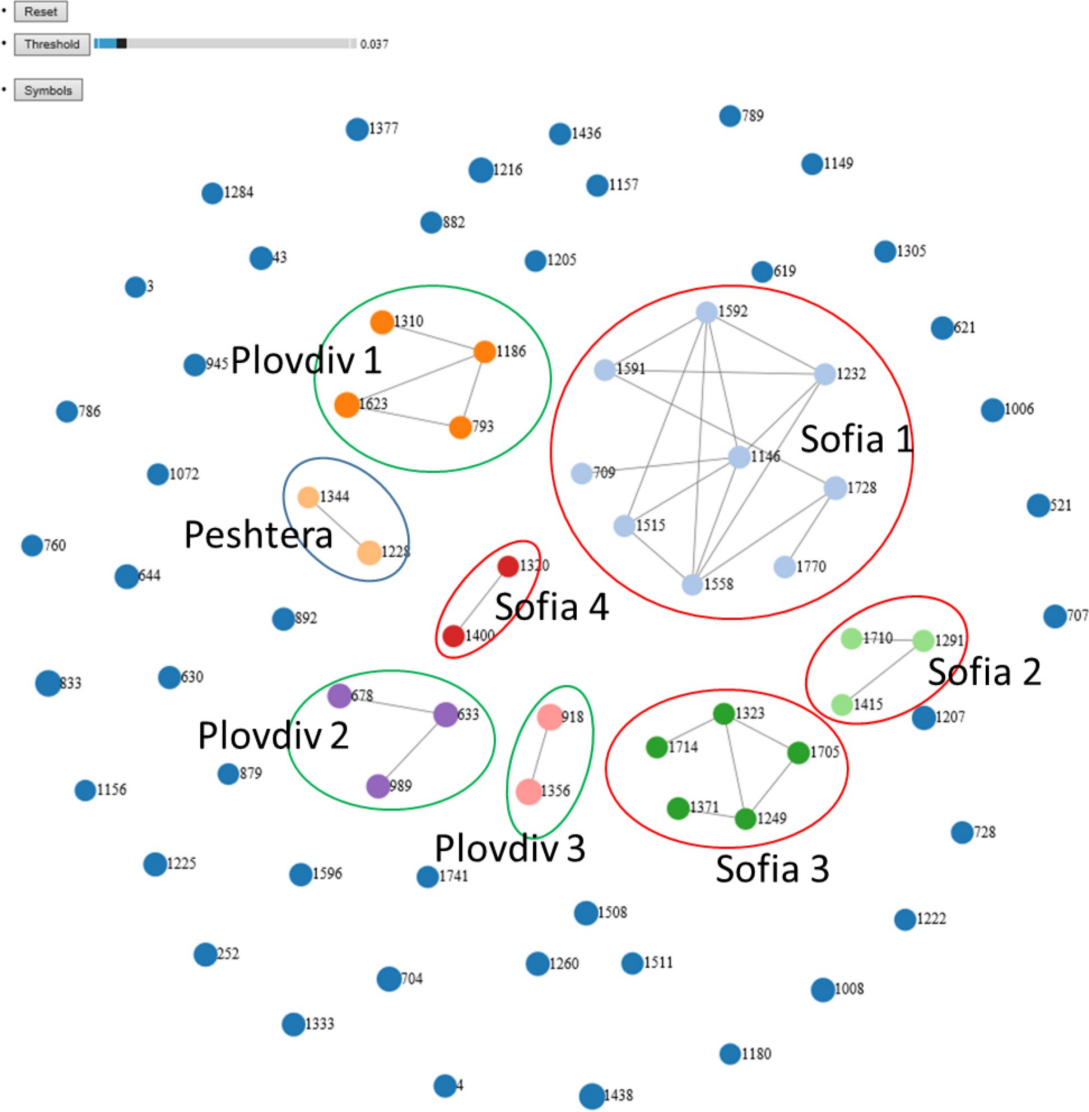
Transmission networks. Nodes represent HCV infected persons. Cases sharing an HCV strain are shown as 2 nodes linked by line (transmission link). Dark blue unlinked nodes–unrelated cases; Clusters are identified by the node color and encircled—Sofia 1(n = 9, genotype 1a)—light blue; Sofia 2 (n = 5, genotype 1a)—dark green; Plovdiv 1 (n = 4, genotype 1a)—dark orange; Sofia 3, 1a (n = 3, genotype 1a)—light green; Plovdiv 2, (n = 3, genotype 3a)—purple; Plovdiv 3, (n = 2, genotype 3a)—pink; Sofia 4, (n = 2, genotype 1b)—red; Peshtera (n = 2, genotype 1b)—light orange.

NGS reads from 72 cases passed all quality filters as implemented in GHOST [23]. The data were used to generate a transmission network and the results are shown in Fig 3. We found eight clusters from three cities. In Sofia, three clusters of cases infected with genotype 1a strains, Sofia 1 (n = 9), Sofia 2 (n = 5) and Sofia 3 (n = 3), and one cluster of cases infected with an HCV genotype 1b strain, Sofia 4 (n = 2), were identified. In Plovdiv, three clusters were found, with cluster Plovdiv 1 (n = 4) being infected with an HCV genotype 1a strain and two clusters, Plovdiv 2 (n = 3) and Plovdiv 3 (n = 2), with genotype 3a strains. In Peshtera, only one cluster, Peshtera 1 (n = 2), of cases sharing an HCV genotype 1b strain was found. Thus, testing of a small sample (n = 72) of HIV-HCV-infected residents of 3 cities identified 30 cases who belong to HCV transmission clusters. The detection of 42% of all tested persons linked to several city-specific transmission clusters indicates a very high rate of HCV transmission among potentially large high-risk communities established in each city. The cases infected with the rare genotypes 2 and 4 were not involved in any of the transmission linkages.

### Strain grouping

**Fig 4:**
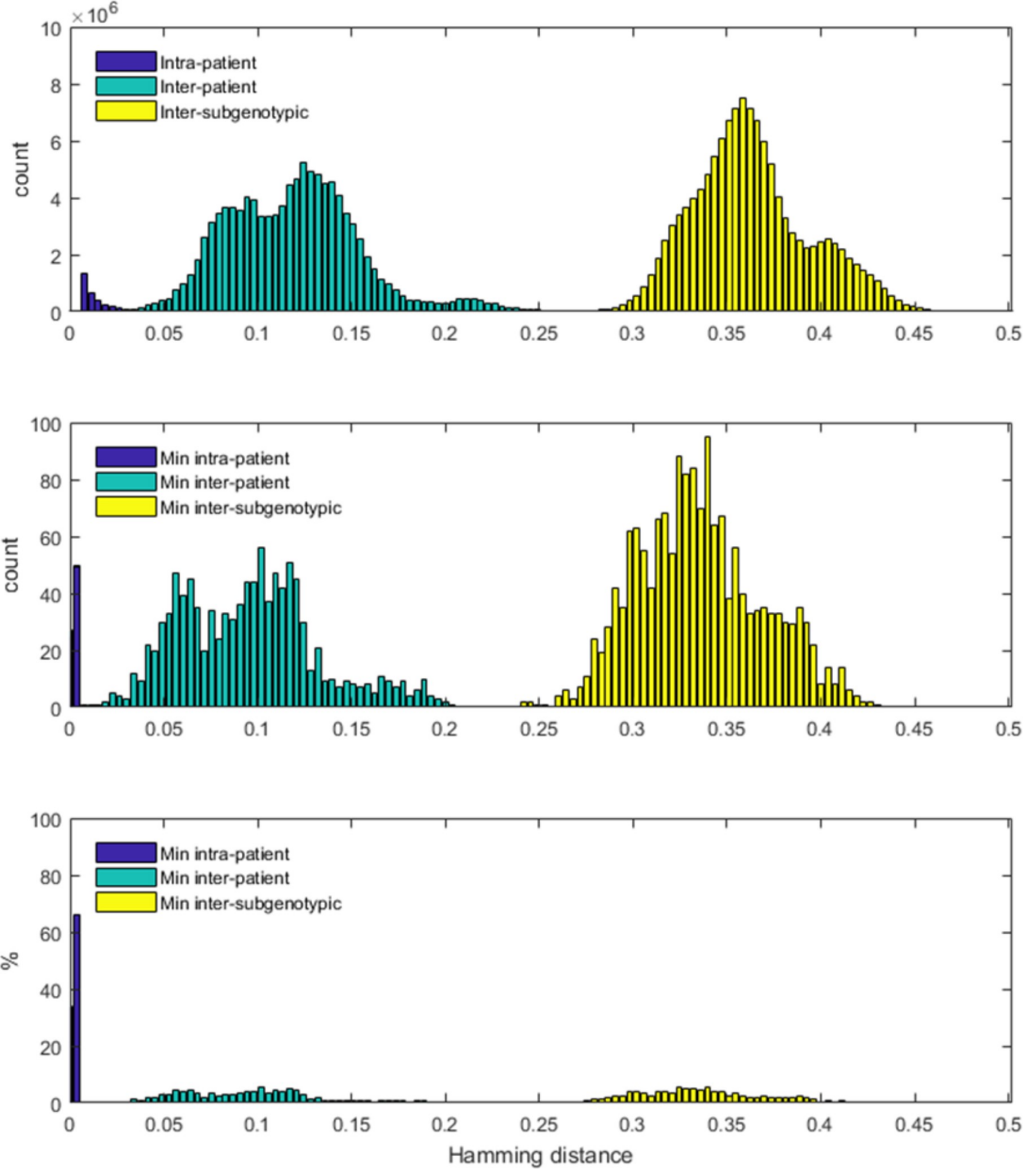
Genetic distances between and within patients and subtypes. Histograms of pairwise Hamming distances among samples. In all panes, the x-axis displays the distance, and the y-axis displays the count/percentage of pairs found to have that distance. Distances within a patient are denoted by blue; distances between patients from the same subgenotype are denoted by green; distances between patients from different subgenotype are denoted by yellow. (A) All distances between the sequences in the three groups. The y-axis represents the count of the pairs found at a certain Hamming distance. (B) Minimum pairwise distances in the three groups. For each pair of patients in the group, the minimum distance is defined as the minimum distance between the sequences of the two patients. The y-axis represents the count of the pairs with this minimum Hamming distance. (C) Percentage of the minimum distances between patients in the three groups respective to all minimum distances found in the group.

Analysis of minimal genetic distances among intra-host HVR1 variants from different persons showed several HCV strains separated by distances, which only slightly exceed the GHOST threshold (0.037). The frequency distribution of these distances is shown in Fig 4.

**Fig 5:**
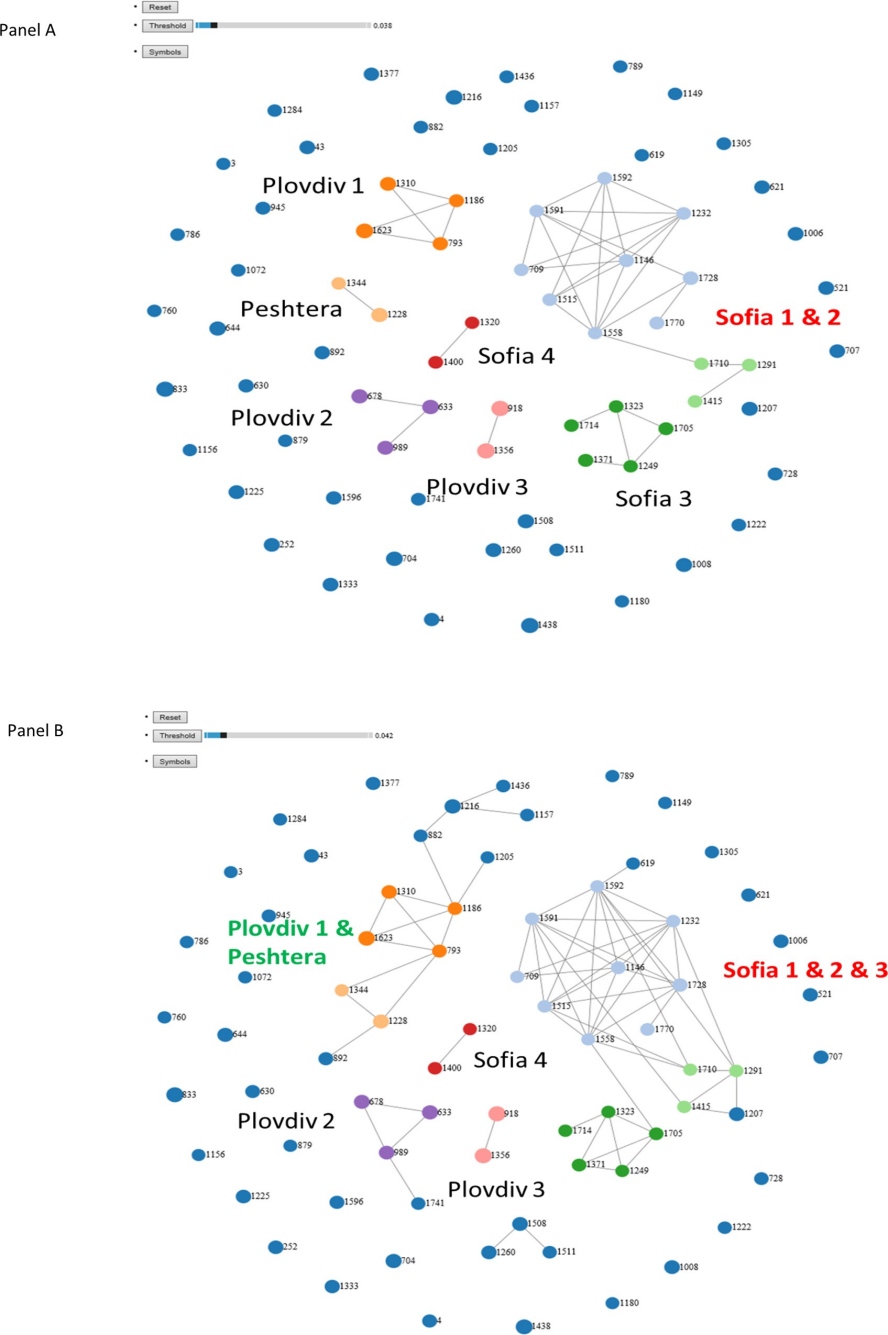
Grouping of cases at genetic distances slightly exceeding the GHOST threshold. The newly linked members, colored in dark blue, reside in the same geographic location as the corresponding transmission clusters. (A) Threshold distance of 0.0378. (B) Threshold distance of 0.042.

At distance of <0.0378 (Fig 5A), transmission clusters Sofia 1 and Sofia 2 became grouped through cases 1558 and 1710, indicating a tight genetic proximity between these 2 transmission clusters. At distance of <0.042, Sofia 3 joined Sofia 1 and Sofia 2 and the group added two new cases for a total of 19 cases. The 2 new cases resided elsewhere but had a history of incarceration in Sofia. Similarly, at distance of <0.042(Fig 5B), Plovdiv 1 was grouped with the Peshtera cluster via case 793, who had coinfection with HCV genotype 1a and 1b strains. This new group was joined by 5 cases from Plovdiv. Significant regrouping of transmission clusters and unlinked cases at the genetic distances slightly exceeding the GHOST threshold indicates a long epidemic history of transmission of a few ancestral strains in each city and an extensive inter-community transmission among the cities.

### K-step networks

**Fig 6:**
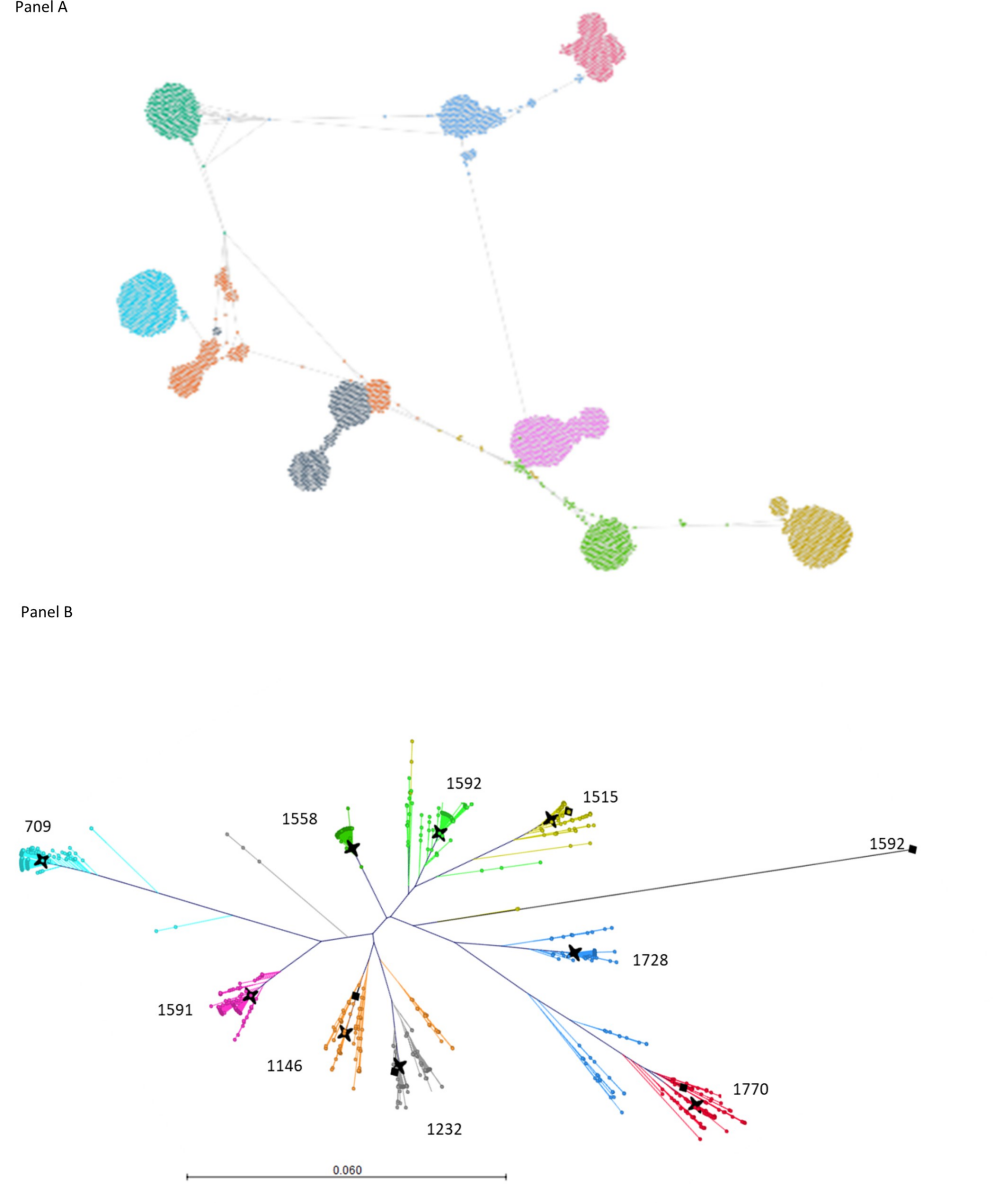
K step network (A) of the Sofia 1 cluster of transmission defined by threshold distance of 0. 037. Phylogenetic tree (B) of the Sofia 1 cluster with all haplotypes with frequency>10. Sanger consensus sequences -black star; major sequence haplotype -black diamond. For cases 709, 1558, 1591 and 1728 the major coincided with the Sanger consensus; the coloring of the cases is the same for both panels.

To analyze genetic relationships among HCV strains from the GHOST-identified transmission clusters, we constructed k-step networks of intra-host HCV HVR1 variants sampled from patients involved in each cluster (Fig 6A). Each patient is infected with many HCV variants organized in a single or a few closely related subpopulations. An important feature of the networks is existence of intermediate HCV variants located between major subpopulations from different persons. In many cases, these minor intermediate variants define the shortest genetic distances between HCV variants from individuals involved in transmission clusters, and GHOST detects transmission links using these distances.

### Consensus sequence of intra-host HCV HVR1 population

For 40 (55.5%) cases, the exact Sanger consensus sequence was not found among intra-host variants detected by NGS but only mapped in phylogenetic proximity to the major variants as exemplified in Fig 6B for cases from the Sofia 1 cluster.

## Discussion

The cohort described in this study is comprised of individuals who have high-risk behavior and are HIV-positive [2]. All studied cases (n = 125) are HIV/HCV coinfected. Coinfections with 2 different blood-borne viruses is frequent among high-risk populations as compared to the general population [24, 25]. Indeed, ~86% of the cases belong to PWID, MSM and sex workers. Genetic analysis shows infections with many HCV strains that belong to 4 genotypes and 5 subtypes, indicating numerous independent introductions of HCV strains to the studied communities. Some HCV strains, however, seemed to be introduced earlier than other. Phylogenetic analysis identified a few clusters of NS5B and HVR1 sequences, suggesting that ancestral strains from each cluster established themselves earlier than HCV strains represented with single sequences scattered across the tree outside of the clusters. The earlier introduction allowed accumulation of genetic differences in progeny HCV strains, which were widely sampled during the specimen collection. A long history of the infections is also supported by observation of long tips in phylogenetic clusters, indicating that at least some HCV strains extensively evolved in the studied population [26].

The detection of transmission clusters was conducted using GHOST. The graphical GHOST output presents transmission networks generated using NGS data [23]. In the networks, nodes represent infected individuals linked together if genetic distance between intra-host HCV HVR1 populations is below a certain threshold [15, 19]. GHOST detected a network with eight transmission clusters. All clusters were comprised of HCV strains of the same subtype, i.e., 1a, 1b or 3a. Each cluster was associated with one of three cities (Sofia, Plovdiv or Peshtera). Interestingly, two cases infected with genotypes 2k and another 2 with genotype 4d were infected with genetically different strains, indicating that each of these two rare genotypes was introduced to the studied populations independently.

However, a long history, suggested by the numerous independent introductions and clustering patterns of HCV strains alone, cannot explain the phylogenetic structure of clusters composed predominantly of very closely related sequences identified by short tips, which is indicative of a recent population expansion of HCV strains in the clusters [27]. Such expansion can be explained by an extremely high rate of transmission, which may result in rapid evolution. Indeed, identification of 40% of all strains forming 8 city-specific transmission clusters among only 72 cases studied by NGS strongly suggests a frequent HCV transmission among the high-risk populations sampled here. This conclusion is additionally supported by observation of 15% of all cases being infected with >1 HCV strains. It was reported that, owing to replication exclusion [28], superinfection frequently leads to a rapid switch from one HCV strain to another [29, 30]. Coexistence of two HCV strains in a single host should be transitional and of short duration [31]. Thus, frequent identification of mixed infections suggests a very high rate of transmissions in a population of hosts infected with several HCV strains.

Analyses of k-step networks of HCV variants from each transmission cluster and distribution of minimal genetic distances among HCV strains circulating in all 3 cities offer a further support to the high-rate of transmission and the resulting rapid HCV evolution. Identification of minority HCV HVR1 variants at a very short distance from intra-host HCV populations from other persons visualized by k-step networks within transmission clusters suggests that HCV was captured in the evolutionary transition from one intra-host subpopulation of HVR1 variants to another. A similar transition, but at the level above strain, was detected by grouping HCV variants using minimal genetic distances between intra-host variants from different persons slightly exceeding the GHOST threshold used for the identification of HCV transmission clusters [15, 20]. Both examples show that, despite a limited sampling from high-risk groups in three cities in the country, genetic analyses detected many HCV strains rapidly evolving from their recent ancestral state. Such detection of transitional viral states seems to be only probable from human population extensively infected for a very long time and experiencing a high-rate of transmission, which results in prevalence of HCV strains existing at different stages of evolution away from their ancestral states. Considering the HIV-HCV coinfection, it is reasonable to suggest that all cases studied here are at extremely high risk of infection with blood-borne infections and may represent a “core” of transmission networks existing among the three cities. Testing HCV strains circulating in such core should facilitate the detection of transitional states of HCV evolution. The identification of >40% of the cases in transmission clusters strongly supports this supposition. Conversely, the detection of HCV strains, genetic distance among which slightly exceeds the GHOST threshold, can be viewed as indicative of membership in a high-risk transmission network.

The cases studied here are not truly representative of the country’s high-risk population in general. Rather, these cases seemingly represent a fraction of the population at an especially high risk of acquisition of blood-borne infections and HCV in particular. This conclusion is supported by the detection of HIV-HCV coinfections, individual infections with >1 HCV strains and 8 HCV transmission clusters spread over 3 cities. This group captured many HCV strains, with some of them circulating over a long time. Limitation of the study is the lack of HCV NGS data from the general population. This is the first molecular epidemiological investigation indicating a long and complex epidemiological history of HCV infections among high-risk populations in Bulgaria. The public health implications of the data analysis obtained by GHOST and their interpretation clearly have great potential to inform and put in motion a targeted intervention in the affected populations.

The minimal NGS dataset is available in compressed .zip format as supporting information S1 File.

## Supporting information

S1 Table List of primer names and sequences used in the study. (DOCX)

S1 File Minimal dataset of HCV HVR1 haplotype sequences used for the study analyses, generated by Illumina NGS and filtered via GHOST quality controls. (ZIP)
