# Supplementary material for: HCV transmission in high-risk communities in Bulgaria
Source: PLoS One. 2019 Mar 5;14(3):e0212350. doi: 10.1371/journal.pone.0212350 (PMC6400337; doi:10.1371/journal.pone.0212350)
Supplement: S1 Table — (DOCX) [file pone.0212350.s001.docx]

PONE-D-18-27061
HCV transmission in high-risk communities in Bulgaria
PLOS ONE

Journal Requirements:

When submitting your revision, we need you to address these additional requirements.
 
Please ensure that your manuscript meets PLOS ONE's style requirements, including those for file naming. The PLOS ONE style templates can be found at
<http://www.journals.plos.org/plosone/s/file?id=wjVg/PLOSOne_formatting_sample_main_body.pdf> and <http://www.journals.plos.org/plosone/s/file?id=ba62/PLOSOne_formatting_sample_title_authors_affiliations.pdf>

1. In your Data Availability statement, you have not specified where the minimal data set underlying the results described in your manuscript can be found. PLOS defines a study's minimal data set as the underlying data used to reach the conclusions drawn in the manuscript and any additional data required to replicate the reported study findings in their entirety. All PLOS journals require that the minimal data set be made fully available. For more information about our data policy, please see <http://journals.plos.org/plosone/s/data-availability>.
 
Upon re-submitting your revised manuscript, please upload your study’s minimal underlying data set as either Supporting Information files or to a stable, public repository and include the relevant URLs, DOIs, or accession numbers within your revised cover letter. For a list of acceptable repositories, please see [http://journals.plos.org/plosone/s/data-availability#loc-recommended-repositories](http://journals.plos.org/plosone/s/data-availability#loc-recommended-repositories). Any potentially identifying patient information must be fully anonymized. TO DO
 
Important: If there are ethical or legal restrictions to sharing your data publicly, please explain these restrictions in detail. Please see our guidelines for more information on what we consider unacceptable restrictions to publicly sharing data: [http://journals.plos.org/plosone/s/data-availability#loc-unacceptable-data-access-restrictions](http://journals.plos.org/plosone/s/data-availability#loc-unacceptable-data-access-restrictions). Note that it is not acceptable for the authors to be the sole named individuals responsible for ensuring data access.
 
We will update your Data Availability statement to reflect the information you provide in your cover letter.

2. Please include captions for your Supporting Information files at the end of your manuscript, and update any in-text citations to match accordingly. Please see our Supporting Information guidelines for more information: <http://journals.plos.org/plosone/s/supporting-information>.


Additional Editor Comments (if provided):
This is a cross-sectional study of HCV transmission among HCV/HIV co-infected individuals in multiple cities in Bulgaria.  The methods and results are well described but additional details should be added as noted by both reviewers and below:
 
The nucleotide sequence range within HVR1 and NS5B that are targeted should be stated explicitly.

The exact location of the amplicons is included now in the Materials and Methods section line 114.
 
Lines 135-136:  Why were only samples with haplotype frequencies of 5 or more analyzed?  Is there some rationale for not analyzing all samples regardless of haplotype frequency?

This frequency is the automatic cut of value used by GHOST.  The value was established by analyzing deep data using biological clones.
Lines 142-143:  how was the threshold determined?

The referenced articles explain the link between this cut off value established from multiple years of accumulated epidemiological information about true relatedness of linked cases and the genetic make-up of the virus.

For the 4 individuals with discordant HVR1 versus NS5B genotype, were these samples re-extracted and re-amplified to confirm these findings?
 Additional specimen was available from one of them, 1216, where the mixed infection was confirmed. This specimen is listed in Table1. This is now clarified in the text as well, line 169-170.
[Note: HTML markup is below. Please do not edit.]

Reviewers' comments:

Reviewer's Responses to Questions

**Comments to the Author**

1. Is the manuscript technically sound, and do the data support the conclusions?

The manuscript must describe a technically sound piece of scientific research with data that supports the conclusions. Experiments must have been conducted rigorously, with appropriate controls, replication, and sample sizes. The conclusions must be drawn appropriately based on the data presented.

Reviewer #1: Yes

Reviewer #2: Partly

This is addresses further in the responses to the comments made by this reviewer.

2. Has the statistical analysis been performed appropriately and rigorously?

Reviewer #1: Yes

Reviewer #2: Yes

3. Have the authors made all data underlying the findings in their manuscript fully available?

The [PLOS Data policy](http://www.plosone.org/static/policies.action#sharing) requires authors to make all data underlying the findings described in their manuscript fully available without restriction, with rare exception (please refer to the Data Availability Statement in the manuscript PDF file). The data should be provided as part of the manuscript or its supporting information, or deposited to a public repository. For example, in addition to summary statistics, the data points behind means, medians and variance measures should be available. If there are restrictions on publicly sharing data—e.g. participant privacy or use of data from a third party—those must be specified.

Reviewer #1: No

Reviewer #2: No

The Sequence data are now available at …

4. Is the manuscript presented in an intelligible fashion and written in standard English?

PLOS ONE does not copyedit accepted manuscripts, so the language in submitted articles must be clear, correct, and unambiguous. Any typographical or grammatical errors should be corrected at revision, so please note any specific errors here.

Reviewer #1: Yes

Reviewer #2: Yes

5. Review Comments to the Author

Please use the space provided to explain your answers to the questions above. You may also include additional comments for the author, including concerns about dual publication, research ethics, or publication ethics. (Please upload your review as an attachment if it exceeds 20,000 characters)

Reviewer #1: This is an extremely well-composed report of HCV molecular epidemiology of a convenience sample in Bulgaria.

The methods are appropriately rigorous and the results justify the conclusions. The discussion appropriately places the results and limitations in context.

The only major concern is that this review doesn't see evidence that the primary data have been deposited in a public database; if that's correct, then this seems not to comply with PLoS policy and general expectations for a molecular epidemiology study.

The NGS data are now available as supplemental information in gzip format.

Minor concerns:

a. Lines 66-67 include this (common) misuse of the term quasispecies: "HCV forms a large population of closely related but distinct genetic variants, also known as quasispecies," which could be more accurately stated, "HCV forms a quasispecies, which is a large population of closely related but distinct genetic variants," - the "quasispecies" is the ensemble, whereas individual sequences are variants.

Thank you, the expression was clarified to state:

“Intra-host, the HCV strain infecting an individual forms a large population of closely related but distinct genetic variants, referred to as quasispecies” line 66-67.

b. Figure 4 has 3 panels, but they are not described in the (too brief) caption for that figure.

Added description of the panels, line 404-408

Reviewer #2: Introduction
A reasonable introduction for the general readership of PLoS One, including setting the context of HIV-viral hepatitis coinfections in Bulgaria. There are some minor details in the description of HCV genetics that could be better described.
1. Authors should refer to Smith et al in reference to HCV genotypes / subtypes (line 65) in place of NCBI (which is unreferenced):
Donald B. Smith Jens Bukh Carla Kuiken A. Scott Muerhoff Charles M. Rice Jack T. Stapleton Peter Simmonds (2014). “Expanded classification of hepatitis C virus into 7 genotypes and 67 subtypes: Updated criteria and genotype assignment web resource”, <https://doi.org/10.1002/hep.26744>

Thank you, the reference list is now updated, lines 65 and 319
2. The authors may briefly clarify the term “intra-host” (line 66) considering the general readership of the journal. For example “Within each individual (termed ‘intra-host’), HCV forms a large population of closely related…” may be helpful for later use if the term.

Suggestion incorporated, line 66

3. On line 73 the authors mention that measuring genetic distances “accurately detects HCV transmission”. I feel that the authors should clarify this statement, as phylogenetic analyses cannot comment definitively on transmission events. The authors may want to clarify that phylogenetic analyses are inferred from available data, cannot comment on directionality, and may be complicated by biased or incomplete sampling of transmission networks.

The statement of this sentence is accurate and further detail could be found in the referred paper. Quasispecies analysis can very accurately inform about transmission events. Here we do not make a claim that this distance informs directionality. On that note, directionality can actually be determined with very high probability using quasispecies analysis, but with different algorithm, which was not applied in this study.
4. In the final paragraph of the introduction, I would suggest that the authors describe the aims and hypothesis of the manuscript, rather than the outcomes.

Good point, the text was amended to clarify, lines 87-92 as follows:

This study was aimed at using GHOST to conduct genetic surveillance of HCV infections among a population of HCV-HIV coinfected patients using serum specimens collected from HIV incidence cases identified during 2010-2014 in 15 cities in Bulgaria. As this is a cohort with high risk behavior the analyses was intended to gain insight into the variability of the HCV virus and any potential transmissions in circumstance when the disease incidence itself did not alert to an outbreak situation. This is the first report on the molecular epidemiology of HIV/HCV coinfections in the country.

Methods
The authors provide description of specimens, nucleic acid preparation and amplification, sequencing and network/genetic analyses. There are some clarifications that could be provided.
5. In the section Specimens (line 98), the authors mention samples were collected from newly diagnosed HIV cases in Bulgaria, 2010-2014 (n=503). It would be helpful to clarify the total number of newly diagnosed cases in Bulgaria during this time. I.E. what fraction of the newly diagnosed population were sampled?

Serum samples were available from 53.8% (n=503) of all newly diagnosed HIV cases (n=934) identified during 2010-2014 in Bulgaria {2}.
6. Can the authors provide information about the participant recruitment sites used for the study? For example, predominately tertiary care facilities etc? This may bias the sampling of the epidemic.

The collection is a representative nationwide study, as of 2014 there were 2077 identified cumulative cases of HIV {2}.
7. Line 99, I believe “questioner” should be “questionnaire”

Corrected, thank you.

8. For the data collected from participants (line 100), what is the time-frame for time-variant variables, e.g. foreign travel, injection drug use? I.e. ever, past 12 months, past six months, past one month?

…questionnaire about sex, age, origin of birth, partners (spouse or cohabitation), occupation, and if they have ever traveled to a foreign country, used injection drug or been imprisoned.
9. Can the authors clarify “partners” on line 100? Do they refer to sex partners? Is it gender of sex partner, number of sex partner? Again, in what time frame?

…questionnaire about sex, age, origin of birth, partners (spouse or cohabitation), occupation, and if they have ever traveled to a foreign country, used injection drug or been imprisoned.

10. It would be helpful to have a flow diagram of participant inclusion/exclusion that matches this section in Methods and Results. I.e. 503 collected ◊ XX HCV ab-positive ◊ XX HCV RNA positive ◊ 125 HCV genotyped ◊ 72 NGS complete. It would be helpful to see where potential participants were lost.

Anti-HCV positive samples (n=203) were tested for HCV RNA using Amplicor Hepatitis C virus Test, version 2.0 (Roche Molecular Systems, Branchburg, New Jersey, USA) [2]. HCV RNA positive specimens (n=171) were genotyped in this study (n=125) and were used for further analysis.

11. In line 107 the authors mention that HCV RNA positive specimens that were genotyped were used in the study. Can the authors clarify why genotyping was necessary for this? Do they consider potential for bias by excluding specimens that were HCV RNA positive but where genotype was not available?

Genotyping is a very good initial indicator of the heterogeneity of a population. NAT testing products that yielded consensus sequence, i.e. were typable, were used further for NGS.

12. In line 132, the authors mention that after passing filters, 20 000 reads randomly selected for further processing. Can the authors clarify in the methods that this was per specimen?

Done

13. Considering the increased immunological pressure and thus high diversity of the hypervariable region, do the authors consider that a threshold of 0.037 may be too high? I appreciate that it is based on previous data, but evolutionary pressures on this region may result in convergent evolution not accurately reflecting shared ancestry. I would suggest the authors consider more stringent sensitivity analyses (perhaps to 0.0102 reported in Campos et al).

The threshold used is indeed based on a wealth of combined epidemiological and molecular data confirming or rejecting relatedness, accumulated over 2 decades. HIV coinfection has been observed to somewhat limit HCV variability in cases of co-infection. Analysis was performed at various levels of stringency, both below and above 0.037. This transmission linkage cut of is the pre-set value in the GHOST informatics pipeline and includes the standard deviation of the distances. At value 0.0102 (the average minimum distance) large number of links are falsely removed.

Results
14. At the start of this section, it would be helpful to first describe the population being studied. As mentioned previously, it would be helpful to include a flow diagram of inclusion / exclusion of individuals through the process. Additionally, it would be useful to describe the characteristics of participants, in total and stratified by inclusion (n=72) and exclusion (n=?) in the study.

The studied population was appropriately described in Materials and methods. Inclusion/exclusion parameters in the study were only guided by sample volume availability, successful amplification, library preparation and generation of NGS data that were of acceptable quality to pass GHOST standards.

15. In line 165 the authors detail the genotype distribution of the NS5b and HVR1 Sanger sequences. Please clarify the numerator in this case (i.e. 72 mentioned in Methods?)

125 were the genotyped RNA positive samples as listed in line 108, 72 were the ones with available good quality NGS data.
16. In line 171 monophyletic clusters are discussed based on HVR1 and NS5b sequences. However, there is no mention of the methods used for this analysis. Were these based on Sanger sequencing? If so, this is not sufficiently described in the Methods section. (Line 128 only mentions that phylogenetic trees being generated using MEGA6, but does not including the model setting, assessment of bootstrap reliability or cluster threshold).

Both HVR1 and NS5b trees are shown in Figure 2 as stated in the text. Bootstrapping is automatically done at 1000 iterations, however since the phylogenies are not particularly useful to asses relatedness by transmission, this value was not considered of great relevance. In this particular instance the bootstrap values for the mentioned clusters were greater than 80%, which is now reflected in the text to the figure.

17. Similarly, on line 172 the authors mention that 85% of genotype 1a sequences generated formed one cluster, but it’s uncertain what region / method was used for this clustering.

Gene name is in the text.
18. On line 174 the authors mention that the “ancestral strain for the largest cluster was most probably one of the first to be introduced in this population”, however there is no mention of how the ancestral strain was generated. How did the authors determine that the ancestral strain of the sequences obtained was ever present in Bulgaria?

Such was not generated, the text simply refers to the possibility that there was such one for each cluster, since the genetic distances between the clusters are large enough to represent different stains that can’t easily evolve into each other.

19. In the next sentence, the authors mention that long tips in NS5B clusters of genotype 1a suggest a long history of the genotype in the population. However, equally long branch lengths are seen for genotype 3a. I do not feel that either of these claims are supported by the data, but rather are hypothesised by the authors. Theories about ancestral strains should be examined using appropriate methods, such as phylodynamic analyses.

Correct, this statement is a hypothesis and this is now clarified in the text.

Presence of long tips in the NS5B clusters of HCV (Fig. 2) indicates a potentially long history of both clusters in the population; while identification of many tightly related NS5B sequences in the clusters suggests a recent expansion of the HCV 1a strains.

20. On line 182, the number of total reads are reported, but not the number of individuals as input (I calculated as 89?). I would suggest the authors include this here (and in participant flow diagram).

21. In line 190 the resolution of discordant genotyping was reported. It would be good to clarify the example of case 793 being initially genotyped using Sanger. E.g. “For example, for case 793, Sanger sequencing of NS5b resulted in subtype 1a and Sanger sequencing of HVR1 revealed subtype 1b”.

Corrected

For example, for case 793, Sanger sequencing of NS5b subtyped it as 1a and Sanger sequencing HVR1 revealed 1b.

22. In lines 197 and 209-2010 the authors report that 15% co-/superinfection and 42% clustering suggests high rate of HCV transmission. I would consider this a discussion of the results, and suggest the authors leave for the Discussion section. Additionally, the authors are lacking additional analyses that may actually inform rate of HCV transmission. Phylodynamic analyses may be able to calculate the reproduction number (R0) of the clusters, to allow estimation of the transmission rate. As these were not calculated I would suggest the authors reframe from hypothesising the rate of HCV transmission in this context.
 We acknowledge the redundancy and left the statement only in the discussion section. The rate of HCV transmission is only referred to as high, since the studied population was not selected in outbreak settings.

Tables and figures
23. Table 1 is not sufficiently detailed to allow interpretation without referencing the text. This table needs to be refined considerably.

Added more detail to the table.

24. For figure 2a, the nucleic acid variation scale is not provided. Also, it is mentioned that reference sequences are obtained from North America. Would it be more appropriate to include European reference sequences to provide more appropriate epidemiological context?

European references would certainly be appropriate, however we rather wanted to use our own well curated database sequences and specifically include cases with known HIV/HCV co-infection.

25. In Figure 3 and Figure 5, “Sofia 4” is missing.

Added, thanks.
26. Figure 4 is not well described. There is no explanation of the three panels, the region being analyzed, nor the method for genetic distance measurement. What are the scales on the y-axis? Could the authors use this data to verify the clustering threshold?

Added better description of the figure and panels:

Genetic distances between and within patients and subtypes.

Histograms of pairwise Hamming distances among samples. In all panes, the x-axis displays the distance, and the y-axis displays the count/percentage of pairs found to have that distance. Distances within a patient are denoted by blue; distances between patients from the same subgenotype are denoted by green; distances between patients from different subgenotype are denoted by yellow.

Panel A:  All distances between the sequences in the three groups. The y-axis represents the count of the pairs found at a certain Hamming distance.

Panel B: Minimum pairwise distances in the three groups. For each pair of patients in the group, the minimum distance is defined as the minimum distance between the sequences of the two patients. The y-axis represents the count of the pairs with this minimum Hamming distance.

27. I’m not sure if the Figure 6A provides additional information to that obtained from phylogenetic analyses. Otherwise, the authors may wish to find a better way to describe the outcome from this figure.

Figure 6A visualizes detailed linkage information using all haplotypes with frequency above 5. Such relatedness may be implied but that is not apparent from the phylogenetic analyses unless the branch lengths are scrutinized. This is affirmed by figure 6B where the Sanger consensus or the major haplotype are quite separated from each other. We added the distance bar to the figure.

28. Figure 5 could be retained in Supplementary Information. There is a large amount of results presented, which may be overly confusing for the readership. I would suggest the authors concentrate on forming an understandable narrative through the manuscript.
 Figure 5 provides linkage by transmission information as it is represented by GHOST. This information is not apparent from the phylogenetic analyses.

Discussion
I have not provided comment on the content of the Discussion as I believe that there is much of the methods and results that requires improvement before further review. My general thoughts of the discussion is that discussion of ancestral strains of HCV is premature without further analysis within the data set. The study is biased by including only individuals recent diagnosed with HIV between 2010 and 2014. There is no description of these individuals in the context HCV in of all of Bulgaria, including the number of people who inject drugs or the number of HIV-positive men who have sex with men. These many be potential sources of HCV not captured in the current study. I would like to point out again concerns regarding the high threshold set for clustering in the HVR1 region. A threshold of 0.037 in this region would likely result in identification of individuals in clusters that do not share reasonably recent ancestors. Finally, there doesn’t seem to be a section of the discussion describing the limitations of the study.

The articles describing the prevalence of HIV, HCV and the incidence of co-infection are referred to in the text. The goal of this study is to examine the presence and molecular epidemiology of HCV in a high risk population, i.e. HIV infected individuals with high risk behavior (MSM, IDU, incarceration). The analysis was intended to evaluate how this epidemiology is explored by GHOST and not necessary to explore the ancestry of the virus. The picture that is revealed is one of multiple introductions from different genotypes and subtypes that are now propagated by transmission.

Limitation of the study is the lack of HCV NGS data from the general population

6. If you would like your identity to be revealed to the authors, please include your name here (optional).

Your name and review will not be published with the manuscript.

Reviewer #1: (No Response)

Reviewer #2: Brendan Jacka


[NOTE: If reviewer comments were submitted as an attachment file, they will be attached to this email and accessible via the submission site. Please log into your account, locate the manuscript record, and check for the action link "View Attachments". If this link does not appear, there are no attachment files to be viewed.]


While revising your submission, please upload your figure files to the Preflight Analysis and Conversion Engine (PACE) digital diagnostic tool, <http://pacev2.apexcovantage.com/>. PACE helps ensure that figures meet PLOS requirements. To use PACE, you must first register as a user. Registration is free. Then, login and navigate to the UPLOAD tab, where you will find detailed instructions on how to use the tool. If you encounter any issues or have any questions when using PACE, please email us at [figures@plos.org](mailto:figures@plos.org). Please note that Supporting Information files do not need this step.

*In compliance with data protection regulations, please contact the publication office if you would like to have your personal information removed from the database.*
